# No substantial long-term bias in the Cenozoic benthic foraminifera oxygen-isotope record

**DOI:** 10.1038/s41467-018-05303-4

**Published:** 2018-07-23

**Authors:** David Evans, Marcus P. S. Badger, Gavin L. Foster, Michael J. Henehan, Caroline H. Lear, James C. Zachos

**Affiliations:** 1 School of Earth & Environmental Sciences, University of St Andrews, St Andrews, KY16 9AL UK; 2 School of Environment, Earth and Ecosystem Sciences, The Open University, Milton Keynes, MK7 6AA UK; 3 Ocean and Earth Science, National Oceanography Centre Southampton, University of Southampton, Southampton, SO17 1BJ UK; 4 Helmholtz Centre Potsdam, GFZ German Research Centre for Geosciences, Telegrafenberg, 14473 Potsdam, Germany; 5 School of Earth and Ocean Sciences, Cardiff University, Cardiff, CF10 3AT UK; 6 Department of Earth and Planetary Sciences, University of California, Santa Cruz, 95064 CA USA

The early Cenozoic (~50 million years ago) was an interval of global warmth that saw cold-blooded reptiles living in the polar regions while mangrove swamps thrived in northern Europe. Foraminiferal oxygen isotope ratios (δ^18^O_c_) represent one of several independent proxies used to place quantitative constraints on palaeotemperature during this ‘greenhouse’ interval. However, the interpretation of geochemical proxy data requires that the possible presence of diagenetic alteration be ruled out or accounted for. To this end, Bernard et al.^1^ conducted laboratory experiments to determine whether solid-state diffusion (SSD) of oxygen between solution and planktonic foraminiferal calcite could impact δ^18^O_c_ of fossil material. Based on a single experiment on recent shells of the planktonic species *Globigerina bulloides*, maintained for three months at 300 °C and 200 bars in ^18^O-pure artificial seawater, the authors found that foraminiferal calcite can exhibit elevated ^18^O/^16^O ratios in the absence of any visible recrystallisation. Using this result, Bernard et al. question the integrity of the deep-sea benthic foraminiferal δ^18^O record^2^. Specifically, they suggest that SSD over ~50 million years could result in a long-term bias of ~1–3‰ in benthic foraminifera δ^18^O_c_, such that high-latitude sea surface temperatures (SST) in the regions of deep water formation at 50 Ma were similar to modern. If correct, this would imply that the early Cenozoic greenhouse was not characterised by a reduced latitudinal SST gradient, and moreover, that deep ocean cooling and ice growth over the Cenozoic have been overestimated. We question these findings based, not least, on two fundamental observations.

**Fig. 1:**
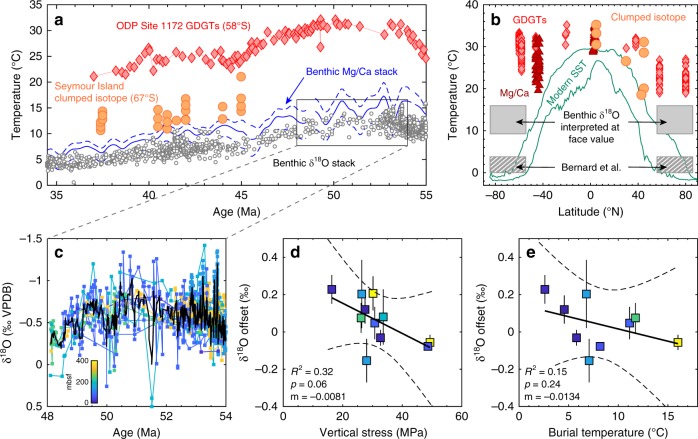
Multiple lines of evidence for early Eocene high-latitude warmth and integrity of the benthic oxygen isotope stack. **a** The Eocene benthic δ^18^O record^2^ interpreted in terms of temperature, assuming δ^18^O_sw_ = −1 ‰ in an ice-free world. The benthic Mg/Ca record^7^ (independent of ice volume) and high-latitude SST proxy data are shown for comparison^5, 13–15^. GDGT abundances are transformed into SST using the TEX_86_^H^ calibration. **b** Early Eocene SST proxy data (48–55 Ma) shown in the context of the zonal range in modern mean annual temperature, demonstrating high-latitude Eocene SST warmth and greatly reduced latitudinal gradient, see ref. ^6^ and references therein. Grey boxes show deep ocean temperatures based on benthic foraminiferal δ^18^O, both interpreted at face value and following Bernard et al.’s SSD model (which implies 0–1 ‰ Eocene-Pleistocene change in δ^18^O_c_ due to cooling, i.e. the Eocene deep ocean was ~0–4 °C warmer than at present). These are plotted as boxes in the high-latitudes as high-latitude SST in the regions of deep water formation cannot be greatly decoupled from the deep ocean. Note that Bernard et al.’s analysis is offset by >15 °C from the other high-latitude SST proxy data, in contrast to the benthic record interpreted at face value. **c** Detail of the benthic δ^18^O record between 48 and 54 Ma (encompassing the long-term warming and cooling trend either side of the Eocene climatic optimum, ~50–52 Ma), with data from different sites coloured as a function of burial depth (metres below sea floor; mbsf). The black line is a 5-point running mean. **d** The mean offset of individual sites shown in **b** from a 5-point running mean through the remainder, plotted as a function of vertical stress ( ± 2SE), based on the present-day water depth and depth in sediment, and the mean density of ocean sediment^16^. Statistics and 95 % confidence intervals are based on 1000 bootstrap simulations including 2SE errors in both variables. The colour scale is the same as in **c**. Regressing the δ^18^O offset against mbsf results in an even less significant slope. **e** The same exercise as in **d**, except shown as a function of burial temperature. There is no significant relationship between geothermal heating and δ^18^O despite an inter-site range in burial temperature of 14 °C. The slope is an order of magnitude smaller than predicted by Bernard et al.’s SSD model. **e** Contains fewer datapoints than **d** because reliable measurements of the geothermal gradient are not available for all sites

First, beyond the warmth suggested by the presence of, e.g. cold-blooded reptiles^3^, alternative quantitative Eocene proxy data from the high-latitude surface ocean can be used as an independent means of assessing the benthic foraminifera δ^18^O record, as the temperature of the deep ocean cannot be greatly decoupled from mean annual SST in the region(s) of deep water formation due to the thermal inertia of water. Crucially, most of these independent proxy systems cannot be susceptible to SSD as they are not based on δ^18^O_c_. Figure 1a shows the Eocene portion of the benthic δ^18^O record interpreted in terms of temperature, assuming that δ^18^O_sw_ in an ice-free world was −1‰. Proxy SST reconstructions based on the relative abundances of Thaumarchaeotal organic membrane lipid molecules (GDGTs) from ODP Site 1172 (~58 °S)^4^, clumped isotopes in shallow-dwelling molluscs from Seymour Island (~67 °S)^5^, as well as multiple other lines of evidence^6^, demonstrate that the Eocene was characterised by high-latitude SST greatly exceeding modern (Fig. 1a, b). Furthermore, deep-ocean temperatures based on benthic foraminiferal Mg/Ca ratios^7^ are within error of the δ^18^O_c_ record for the ice-free early Eocene (Fig. 1a), which would be highly coincidental if both were diagenetically biased. While all proxies have associated uncertainties, the coherence of trace element, isotopic and organic proxy data, all of which reconstruct high-latitude SST 10–20 °C warmer than at present throughout the Eocene, are irreconcilable with deep ocean temperatures similar to today, as suggested by Bernard et al. We stress that these independent proxy datasets are globally distributed (Fig. 1b), underwent very different post-depositional histories, and have widely differing susceptibilities to diagenetic alteration. What is more, recent climate modelling work has shown that a reduction in the latitudinal SST gradient of the magnitude shown by the multiple proxies in Fig. 1b is physically plausible^8^.

Second, if solid-state oxygen diffusion has impacted Eocene benthic foraminiferal calcite, a testable hypothesis is that δ^18^O_c_ from different sites within a given time-slice should exhibit large differences that are well-correlated with burial depth or geothermal heating. The most negative benthic δ^18^O values of the Cenozoic are observed between 48 and 54 Ma, and since these data span 11 different sites, we use this interval to test Bernard et al.’s hypothesis that these negative values are due to burial-induced SSD. Although the 11 sites span a range in maximum (i.e. present day) burial depth of between ~50 and 350 m, there is no visible offset between any individual site and the 5-point running mean through all sites (Fig. 1c). This analysis is quantitatively extended in Fig. 1d, e, which display the mean offset of individual sites from a 5-point running mean through the remainder, plotted as a function of present-day total vertical stress and geothermal heating respectively. No individual site is offset from the running mean by more than 0.2‰ (equivalent to <1 °C temperature bias), despite a range in burial depths of ~300 m and a ~14 °C range in geothermal heating. This is at odds with the SSD calculations of Bernard et al., which would predict an order of magnitude larger (>2‰) range in Eocene inter-site benthic δ^18^O, including very little SSD at the shallowest, coolest sites. We note that the deep ocean is not spatially homogeneous with respect to temperature, Δ[CO_3_^2−^], δ^18^O_sw_, or the prevalence of early-stage diagenetic recrystallisation. Therefore, the minor offset between Eocene sites that we observe may reflect primary geochemical signals.

Based on the above considerations, we demonstrate there is little or no evidence for a substantial temperature/burial depth-dependent SSD-derived bias in the Cenozoic benthic δ^18^O record, and abundant evidence supporting high-latitude, and therefore deep ocean, warmth. Given that Bernard et al. show that SSD can occur in certain situations, the question then becomes: why are the SSD effects expected on the basis of their experimental results not seen in fossil foraminifera? One potential reason is the calcite grain size the authors assumed when applying their SSD model to foraminifera, such that experimental work on core-top specimens may not be an appropriate basis from which to extrapolate to fossil samples. Bernard et al. use a published foraminifer grain size estimate of 50–250 nm to derive an activation energy (Ea) of 82–94 KJ mol^−1^. Crucially, this ignores the fact that early-stage minor diagenetic recrystallisation of benthic foraminiferal calcite likely results in a larger grain size^9, 10^ compared to pristine modern specimens. This is a different process to SSD, which cannot significantly alter the bulk isotopic composition of individual shells as early recrystallisation takes place soon after burial and therefore at a similar temperature to the overlying deep water, resulting in calcite with a similar δ^18^O_c_ to the primary foraminiferal calcite^11^. To our knowledge, there are no published estimates of calcite grain sizes in fossil benthic foraminifera, although previous studies have noted a large diagenetic crystal size in planktonic foraminifera on the ocean floor^9, 10^. If minor recrystallisation results in an increase in grain size to 500 nm, for example, then the Ea applicable to fossil foraminifera would be ~100 KJ mol^−1^ and SSD would impact Eocene benthic foraminifera δ^18^O by <1‰, in good agreement with our analysis (Fig. 1d, e). A crystal size of ~1 µm would mean the applicable Ea is >120 KJ mol^−1^, in which case SSD would be unresolvable in Cenozoic samples. Thus, until the grain size and hence susceptibility of fossil foraminifera to undergo SSD has been fully determined (as opposed to pristine modern samples), the results of Bernard et al. cannot inform us of the potential presence of a long-term bias when interpreting δ^18^O data from Cenozoic carbonates.

Despite the disagreement discussed above, we concur with Bernard et al. that a thorough understanding of diagenetic processes is essential to informative palaeoclimate reconstructions. Indeed, it may be that SSD is important in certain situations and should be considered when interpreting sample data from earlier in the Phanerozoic and Proterozoic. However, within the Cenozoic benthic record we show at most a minor effect of burial depth and geothermal heating on δ^18^O_c_ (Fig. 1c–e), which would not be the case if SSD, as parameterised by Bernard et al, were the cause of significant bias. In addition, numerous other proxies corroborate high-latitude (and therefore deep ocean) Eocene warmth, in good agreement with the benthic δ^18^O stack interpreted at face value. Contrary to the assertion of Bernard et al.’s title, reconciling deep-ocean temperatures similar to today with an ice-free, high-CO_2_ world^12^ would be a greater ‘paradox’ than the current challenges facing the palaeoclimate community.
